# Modulation of Dietary 5-Hydroxymethylfurfural-Induced Intestinal Epithelial Stress by Blueberry Polyphenols

**DOI:** 10.3390/ijms27156665

**Published:** 2026-07-26

**Authors:** Rosario Mare, Francesca Rita Noto, Martina Rago, Kardelen Aslan, Angelo Galluccio, Luana Carmen Mirabello, Ilenia Lio, Samantha Maurotti, Gülhan Samur, Arturo Pujia, Tiziana Montalcini

**Affiliations:** 1 Department of Medical and Surgical Sciences, Magna Græcia University, 88100 Catanzaro, Italy; mare@unicz.it (R.M.); francescarita.noto@unicz.it (F.R.N.); ilenia.lio@studenti.unicz.it (I.L.); pujia@unicz.it (A.P.); 2 Department of Clinical and Experimental Medicine, Magna Græcia University, 88100 Catanzaro, Italy; martina.rago@studenti.unicz.it (M.R.); luanacarmen.mirabello@studenti.unicz.it (L.C.M.); tmontalcini@unicz.it (T.M.); 3 Department of Nutrition and Dietetics, Faculty of Health Sciences, Hacettepe University, 06100 Ankara, Turkey; kardelen_aslan2@hotmail.com (K.A.); gsamur@hacettepe.edu.tr (G.S.); 4 Clinical Nutrition Unit, Renato Dulbecco Hospital, 88100 Catanzaro, Italy; angelo.galluccio@aourenatodulbecco.it; 5 Research Center for the Prevention and Treatment of Metabolic Diseases (CR METDIS), Magna Græcia University, 88100 Catanzaro, Italy

**Keywords:** 5-hydroxymethylfurfural (5-HMF), blueberries, polyphenols, Caco-2 cells, ultra-processed foods

## Abstract

Ultra-processed foods expose consumers to heat-induced contaminants like 5-hydroxymethylfurfural (5-HMF), which impairs intestinal homeostasis via oxidative stress and inflammation. This study quantified 5-HMF in bakery products and evaluated blueberry polyphenols’ capacity to attenuate 5-HMF-induced cellular stress in an in vitro Caco-2 intestinal model. 5-HMF levels in commercial bakery products were determined using a colorimetric assay and HPLC-UV. Differentiated Caco-2 cells were exposed to 5-HMF (0.4 mM) for 48 h, alone or co-treated with blueberry juice (15 µg/mL of phenol equivalents). ROS production, lipid accumulation, gene expression of antioxidant, lipid, and inflammatory markers, and NF-κB/ERK signaling pathways were analyzed. HPLC-UV accurately quantified 5-HMF in bakery products (exceeding 2 mg/100 g), revealing systematic overestimation by the colorimetric method. In Caco-2 cells, 5-HMF significantly increased ROS, lipid accumulation, inflammatory cytokines (NLRP3, IL1B, and IL18), and activated NF-κB and p-ERK. Co-treatment with blueberry juice reduced ROS and lipids, upregulated the NRF2 antioxidant pathway, and drastically suppressed NF-κB -mediated inflammation. 5-HMF induced markers of metabolic dysfunction, oxidative stress, and inflammation in the intestinal epithelial model. Polyphenol-rich blueberry juice attenuated 5-HMF-induced alterations at the tested concentration, suggesting a potential protective nutritional strategy against epithelial stress associated with food-processing contaminants.

## 1. Introduction

Among food-processing contaminants, 5-hydroxymethylfurfural (5-HMF) is a carbohydrate-derived compound that forms during the Maillard reaction and sugar degradation under heat treatment. It is commonly found in thermally processed foods, including bakery products, biscuits, breakfast cereals, honey and several ultra-processed foods (UPFs) [1,2,3]. A high intake of UPFs has been associated with cardiometabolic diseases and gastrointestinal disorders [4,5,6].

The potential harmful effects of 5-HMF on human health are still not completely understood. Although the International Agency for Research on Cancer has not classified 5-HMF as a carcinogen, concerns have been raised about the possible genotoxic and carcinogenic effects of some of its metabolites, such as 5-sulfoxymethylfurfural and 5-chloromethylfurfural [7,8]. Previous in vitro and animal studies have suggested that 5-HMF may affect oxidative stress, inflammation, energy metabolism, and DNA integrity [9,10,11].

Growing evidence also indicates that 5-HMF may interfere with intestinal homeostasis by promoting oxidative stress, inflammatory responses, epithelial barrier dysfunction, and gut microbiota alterations. These mechanisms may contribute to gastrointestinal dysfunction and chronic inflammatory conditions [12,13]. Since the intestinal epithelium is the first site of contact with food-derived compounds, studying the effects of 5-HMF on intestinal cells is particularly important. Differentiated Caco-2 cells are widely used as an in vitro model of the intestinal epithelium because they acquire enterocyte-like features, including polarization, brush border formation, tight junctions, and transport properties [14]. However, data on the effects of 5-HMF in Caco-2 cells, especially regarding oxidative stress and inflammation, are still limited [15].

Polyphenols are bioactive compounds known for their antioxidant and anti-inflammatory properties. Several studies have shown that polyphenols can modulate oxidative and inflammatory responses in intestinal epithelial models, including Caco-2 cells, through pathways such as NRF2, NF-κB, and ERK [16,17,18,19]. Blueberries are rich in anthocyanins and other polyphenols, and their bioactive compounds have been reported to reduce oxidative damage and inflammatory responses in intestinal epithelial cells [20,21,22,23,24]. Therefore, blueberry-derived bioactive compounds may represent a potential nutritional strategy to counteract the harmful effects of food-derived toxic stressors.

Based on these considerations, we quantified 5-HMF levels in selected bakery products frequently consumed as breakfast or snack foods and used these data to support the rationale for an in vitro intestinal exposure model. Furthermore, this study investigated the effects of 5-HMF on oxidative stress and inflammatory response using differentiated Caco-2 cells as an intestinal epithelial model. Finally, we evaluated whether blueberry juice could counteract 5-HMF-induced cellular alterations. To our knowledge, the ability of blueberry-derived polyphenols to modulate 5-HMF-induced oxidative and inflammatory stress in intestinal epithelial cells has not been previously explored. These findings may contribute to a better understanding of the potential intestinal effects of food-processing contaminants commonly present in ultra-processed foods widely consumed and provide preliminary mechanistic evidence supporting the protective role of blueberry-derived bioactive compounds against 5-HMF-induced epithelial stress.

## 2. Results

### 2.1. Chemical Analysis

The 5-HMF extracted from bakery products was quantified by an innovative colorimetric assay and a traditional HPLC technique. Data obtained are listed and compared in Table 1.

The colorimetric detection of 5-hydroxymethylfurfural (5-HMF) via Seliwanoff-based UV-Vis spectrophotometry yielded inconsistent results across the analyzed food extracts. Spectroscopic profiling failed to provide a definitive identification of the resorcinol-HMF chromophore, as the characteristic absorption maxima were often poorly resolved or obscured by background noise. While several extracts produced weak signals, others exhibited anomalous negative absorbance profiles, despite following broader spectral trends. Although most samples recorded absorbance values within the 0.1–1.1 range between 450 and 500 nm, the method demonstrated poor analytical precision. Technical replicates showed high instability, with standard deviations (SD) frequently nearing 50%. Quantitative analysis based on HMF external standards identified Sample #5 as having the highest concentration (>300 ppm), followed by Samples #4 (271.5 ± 0.2 ppm) and #3 (265.6 ± 31.5 ppm). Conversely, Sample #15 showed the lowest detectable levels (32.7 ± 72.5 ppm), while Samples #13, #19, #21, and #26 fell below the limit of detection or yielded negative values (Table 1).

Comparative analysis via HPLC at λ = 484 nm revealed a significant discrepancy compared to direct UV-Vis spectrophotometry. While HPLC identified Samples #17, #14 and #25 as having the highest HMF content (26.82 ± 21.18 ppm; 24.54 ± 16.25 ppm and 19.17 ± 13.94 ppm, respectively), these values were an order of magnitude lower than those estimated by the Seliwanoff spectrophotometric assay. Furthermore, the persistence of high technical variability (SD ~50%) across both methods suggests that instability is inherent to the resorcinol-HMF complex formation rather than a limitation of the detection system itself. The observation of negative results for Samples #1, # 6, #9, and #12 further underscores the analytical challenges posed by matrix-induced interferences.

To assess the accuracy of the colorimetric findings, 5-HMF levels were re-evaluated using a validated benchmark method involving direct HPLC-UV detection of the native molecule at its characteristic maximum wavelength (λ = 285 nm). This gold-standard approach yielded HMF concentrations significantly lower than those predicted by the Seliwanoff-based assay, highlighting a profound discrepancy between the two methodologies.

**Figure 1:**
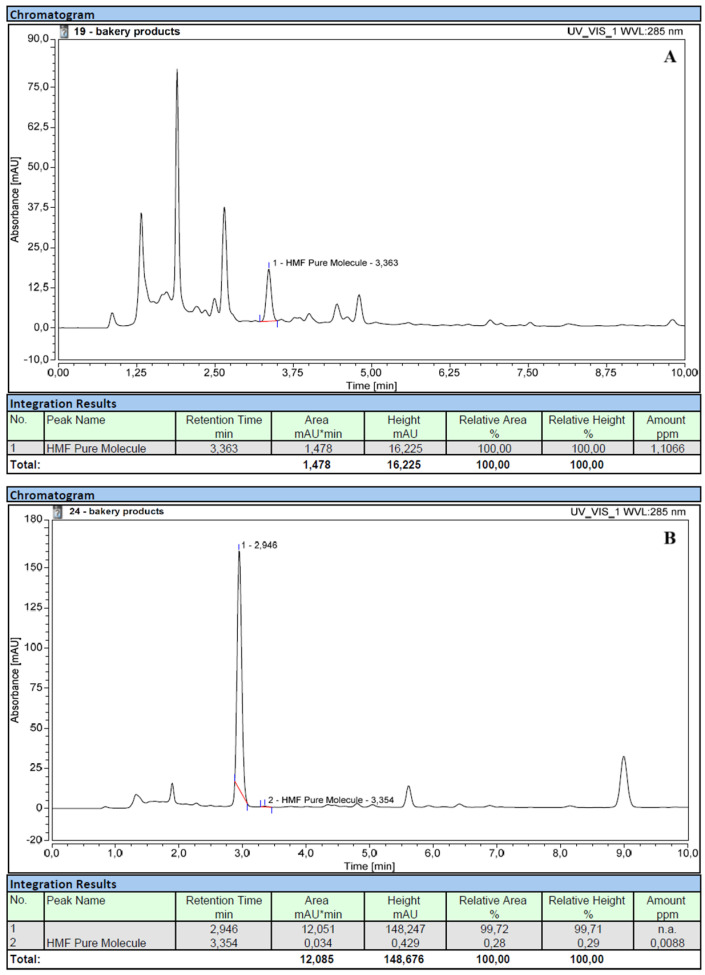
HPLC analyses of 5-HMF in bakery products performed with the traditional technique. Sample #19 yielded the highest value (**A**) while Sample #2 provided the lowest (**B**).

Quantification via direct HPLC provided substantially improved analytical precision. Sample #19, representing whole meal rusks, exhibited the highest 5-HMF concentration (1.103 ± 0.006 ppm), corresponding to 2.205 ± 0.013 mg/100 g of product (Figure 1A), while the minimum detectable level was recorded in Sample #24 (0.009 ± 0.001 ppm or 0.018 mg/100 g; Figure 1B), representing hazelnut breakfast biscuits. These results suggest that the previous colorimetric estimations were heavily influenced by matrix interferences, leading to a systematic overestimation of HMF content.

To evaluate the consistency and reliability of the three analytical methodologies, comparative statistical analyses were performed (Supplemental Figure S1). The Shapiro–Wilk test revealed a normal distribution for the UV-Vis data (*p* = 0.0708), whereas both the HPLC Assay (*p* = 0.0016) and the traditional HPLC approach (*p* = 0.0006) exhibited non-normal distributions (Supplemental Figure S1A). Interestingly, Pearson correlation analyses demonstrated that neither the UV-Vis spectrophotometric data (*p* = 0.23) nor the HPLC Assay values (*p* = 0.259) correlated significantly with the traditional HPLC reference method (Supplemental Figure S1B), highlighting a lack of linear convergence between the alternative methods and the standard approach. Concurrently, comparing the concentration profiles via a one-way ANOVA mixed-effects model confirmed that the three analytical frameworks yielded drastically disparate results (*p* < 0.0001; Supplemental Figure S1C).

**Figure 2:**
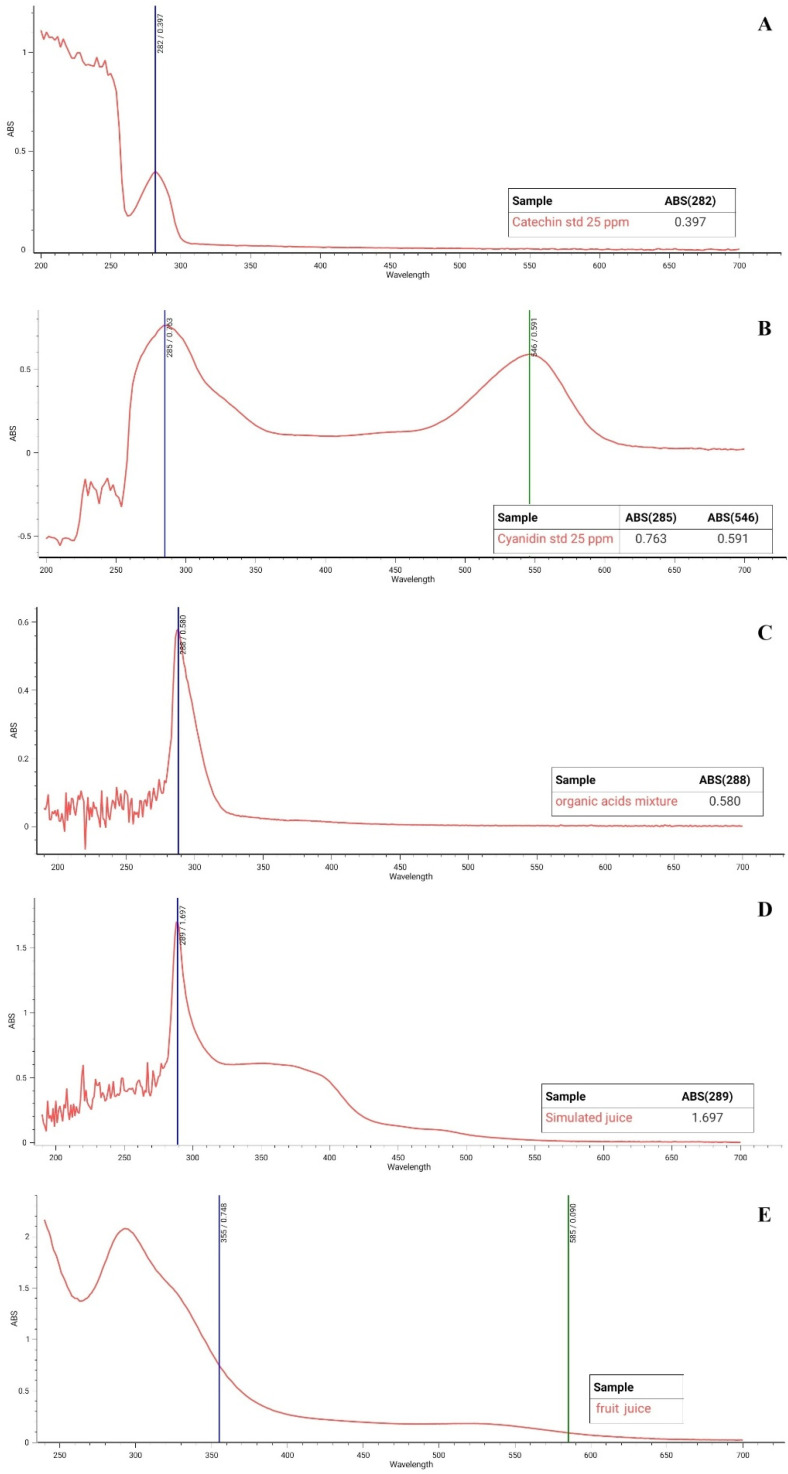
UV-Vis spectrophotometric spectra of catechin (**A**), cyanidin (**B**), organic acids generally contained in blueberry (**C**), a simulated blueberry juice (**D**) and the natural blueberry juice (**E**).

The UV-Vis spectrum of fresh blueberry juice showed absorption bands around 293 nm and 520 nm, which are suggestive of the presence of phenolic compounds and anthocyanin-like pigments (Figure 2E). Interestingly, catechin, cyanidin and a mixture of organic acids generally found in blueberry juice exhibited different UV-Vis spectra when analyzed individually (Figure 2A–C). The mixture of these solutions to partially simulate the composition of the blueberry juice resulted in a change in the main absorbance peak, with a maximum wavelength value close to 290 nm, as for the natural food matrix (Figure 2D and E, respectively).

**Figure 3:**
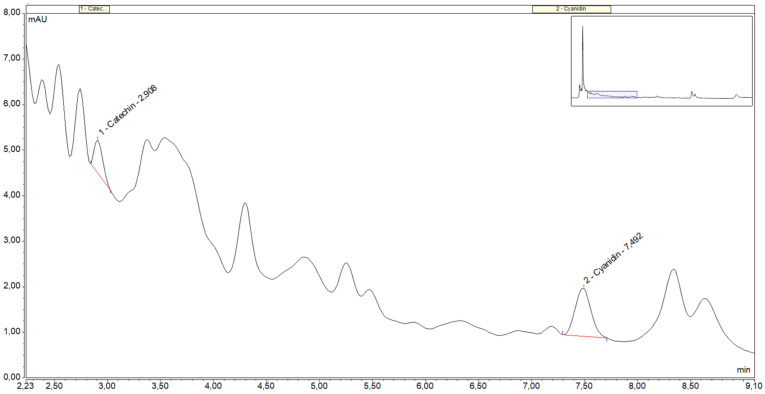
HPLC separation and quantification of catechin and cyanidin contained in blueberries.

Based on these results, the fresh natural juice obtained from blueberries was characterized by colorimetric assay and liquid chromatography, and the results are summarized in Table 2 and Figure 3.

Fresh blueberry juice had a total phenolic compound content of 0.587 ± 0.015 mg equivalent to gallic acid. In addition, there was a concentration of flavonoids, including cyanidin, of ~44 ppm, which overall were shown to exert an antioxidant and free radical-inhibiting power of approximately 31%I (Table 2).

HPLC provided in-depth details about the UV-vis spectrophotometric results. Specifically, chromatograms of the blueberry juice obtained by manually squeezing revealed the presence of catechin and cyanidin, with retention times of approximately 2.9 min and 7.5 min, respectively. The corresponding concentrations were determined to be 0.699 ± 0.014 ppm for catechin and 3.456 ± 0.010 ppm for cyanidin (Figure 3).

### 2.2. Blueberry Juice Attenuates 5-HMF-Induced Oxidative Stress and Inflammatory Response in Caco-2 Cells

Before evaluating oxidative and inflammatory responses, cell viability was assessed by MTT assay in differentiated Caco-2 cells exposed to 5-HMF at 0.2 and 0.4 mM, either alone or in combination with blueberry juice. Neither 5-HMF alone nor the combined 5-HMF/blueberry treatment reduced cell viability at the tested concentrations, indicating the absence of overt cytotoxicity. Blueberry co-treatment did not impair cell viability and, at 0.4 mM, increased the MTT absorbance signal. These data supported the use of 0.4 mM 5-HMF as a non-cytotoxic experimental condition to investigate early epithelial stress responses (Supplemental Figure S2).

**Figure 4:**
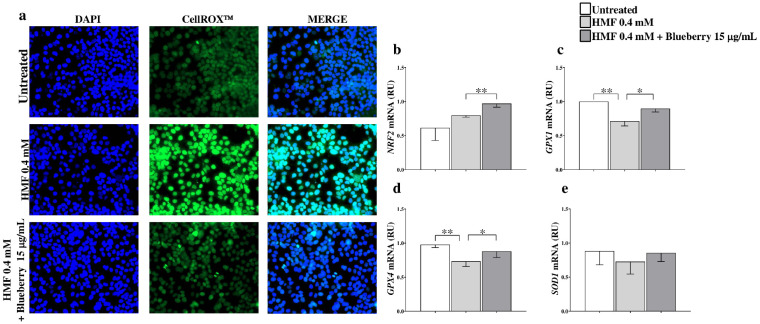
Blueberry juice attenuates 5-HMF-induced oxidative stress response in Caco-2 cells: (**a**) Representative fluorescence images of intracellular ROS production detected by CellROX™ (Green) in untreated cells, cells treated with HMF (0.4 mM), and cells co-treated with HMF and blueberry (15 µg/mL). Cell nuclei were counterstained in blue (**b**–**e**) Relative mRNA expression levels of antioxidant and detoxification genes (NRF2, GPX1, GPX4, and SOD1) were measured using Real-Time PCR. Data were analyzed using the 2^−^ΔΔCt^ method and normalized to β-ACTIN. Data are represented as mean ± SD of three independent experiments. *p*-values calculated by Student’s *t*-test: * *p* < 0.05, ** *p* < 0.01. Abbreviations: RU = relative unit.

Differentiated Caco-2 cells were treated with HMF alone or in combination with blueberry juice to evaluate whether blueberry juice could attenuate HMF-induced cellular stress (Figure 4). HMF treatment significantly increased intracellular ROS production, indicating the induction of oxidative stress (Figure 4a). HMF did not significantly affect NRF2 mRNA expression, whereas it reduced GPX1 and GPX4 mRNA levels (*p* < 0.01, respectively). Co-treatment with blueberry juice reduced ROS levels and significantly increased NRF2 mRNA expression compared with HMF alone (*p* < 0.01), while partially restoring GPX1 and GPX4 mRNA expression (*p* < 0.05, respectively). No significant changes were observed in SOD1 mRNA expression in all the treatments (Figure 4b–e).

**Figure 5:**
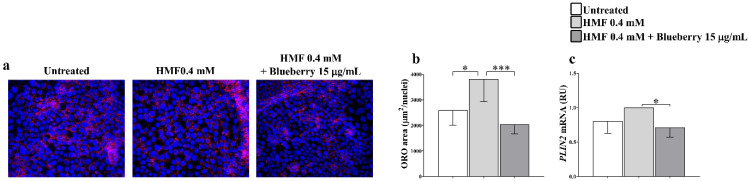
Blueberry juice attenuates 5-HMF-induced neutral lipid accumulation in Caco-2 cells: (**a**) Representative images of lipid accumulation in Caco-2 cells. (**b**) Neutral lipid accumulation in Caco-2 cells was assessed using Oil Red O staining (Red). Cell nuclei were counterstained in blue. (**c**) Relative mRNA expression levels of the lipid accumulation marker (PLIN2) were measured using Real-Time PCR. Data were analyzed using the 2^−^ΔΔCt^ method and normalized to β-ACTIN. Data are represented as mean ± SD of three independent experiments. *p* values calculated by Student’s *t*-test: * *p* < 0.05, *** *p* < 0.001. Abbreviations: RU = Relative unit.

HMF also increased neutral lipid accumulation and PLIN2 mRNA expression (*p* < 0.05), suggesting an alteration of lipid storage and cellular metabolic homeostasis. Blueberry juice co-treatment reduced both neutral lipid accumulation and PLIN2 expression (*p* < 0.001 and *p* < 0.05, respectively), indicating a protective effect against HMF-induced lipid accumulation (Figure 5).

**Figure 6:**
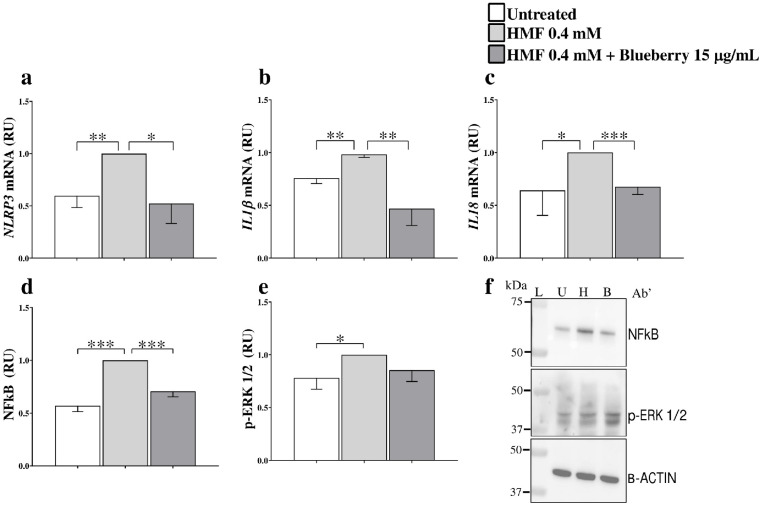
Blueberry juice attenuates 5-HMF-induced oxidative stress and inflammatory response in Caco-2 cells: (**a**–**c**) Relative mRNA expression levels of inflammation markers (NLRP3, IL1β, and IL18) were measured using Real-Time PCR. Data were analyzed using the 2^−^ΔΔCt^ method and normalized to β-ACTIN. (**d**–**f**) Protein levels by Western blotting and relative quantification of p-Erk1/2 and NF-κB expression. Data are represented as mean ± SD of three independent experiments. *p* values calculated by Student’s *t*-test: * *p* < 0.05, ** *p* < 0.01; *** *p* < 0.001. Abbreviations: RU = relative unit; L = ladder; U = untreated; H = HMF 0.4 mM; B = HMF 0.4 mM + Blueberry 15 µg/mL.

Moreover, HMF induced a pro-inflammatory response, as shown by the increased mRNA expression of NLRP3, IL1B, and IL18, together with increased NF-κB protein expression (*p* < 0.01, *p* < 0.01, *p* < 0.05 and *p* < 0.001, respectively; Figure 6a–d). HMF also increased p-ERK protein levels compared with control cells (*p* < 0.05). Blueberry juice reduced the HMF-induced increase in inflammatory markers and NF-κB expression (*p* < 0.001). In addition, blueberry juice showed a trend towards reducing HMF-induced ERK phosphorylation, although this effect did not reach statistical significance (*p* = 0.07) (Figure 6). These results suggest that HMF induces oxidative stress and inflammatory activation in differentiated Caco-2 cells, while blueberry juice exerts a protective effect against HMF-induced cellular stress.

## 3. Discussion

Modern eating habits have progressively shifted towards a higher consumption of prepackaged, heat-processed, and ultra-processed foods, including biscuits, rusks, snacks, breakfast products, and processed fruit-based beverages. These products may represent relevant dietary sources of food-processing contaminants, such as 5-HMF, a heat-induced compound mainly formed during the Maillard reaction and sugar degradation. Its formation is influenced by sugar content, thermal treatment, storage conditions, water activity, and food matrix composition [3,25]. This structural and processing dependency is clearly reflected in our screening of commercial baked goods (Supplemental Table S1). While standard biscuits and cookies, typically baked at 170–180 °C up to 15 min, exhibited marked variability due to different raw material formulations, the highest 5-HMF accumulation was found in matrices subjected to extended or dual thermal processing. Specifically, whole meal bread rusks (Sample #19), which undergo a double baking and toasting process, reached the maximum concentration (2.205 ± 0.013 mg/100 g). This sharp increase aligns with kinetic models showing that double-baked products face a drastic drop in water activity, which exponentially accelerates 5-HMF formation in the carbohydrate-rich crust [26,27]. Conversely, hazelnut breakfast biscuits (Sample #24) registered the lowest detectable levels (0.018 mg/100 g), confirming that specific ingredient formulations, such as the incorporation of lipid-rich nuts or alternative sugar profiles, can drastically modulate or mitigate the non-enzymatic browning cascade. Therefore, evaluating both the occurrence of 5-HMF in commonly consumed bakery products and its biological effects on intestinal epithelial cells is relevant to better understand its potential impact at the gut level.

In addition, a healthier dietary approach should include not only limiting excessive consumption of heat-processed and ultra-processed foods but also promoting fresh foods rich in antioxidant bioactive compounds. This is consistent with the Italian Dietary Guidelines, which recommend increasing fruit and vegetable consumption and limiting foods rich in free sugars, including sweets and sugar-sweetened products [28].

Our chemical analysis confirmed the presence of 5-HMF in the analyzed bakery products, in agreement with previous studies reporting variable levels of this contaminant in thermally processed cereal-based foods. Ramírez-Jiménez et al. reported HMF concentrations in selected bakery products [29], while Petisca et al. confirmed the presence of HMF and furfural in commercial bread and bakery products [30]. Overall, our data support the concept that baked foods frequently may contribute to dietary exposure to 5-HMF, although its amount can vary widely depending on formulation and processing conditions [2,26].

Based on our chemical screening results, we established a physiologically relevant rationale for the concentration of 5-HMF utilized in our cellular models. Considering that the standard Italian dietary portion (LARN) for sweet baked products is set at 30 g, the ingestion of products with high contamination levels, such as the whole meal bread rusks examined in this study (Sample #19, 2.205 mg/100 g), would lead to a localized exposure in the gastrointestinal lumen [31,32]. Therefore, the concentration of 0.4 mM was specifically selected to mimic an overconsumption or high-exposure dietary scenario. This dosage allows for the assessment of early intestinal epithelial stress markers, metabolic alterations, and inflammatory priming before triggering extensive cell death or absolute cytotoxicity, which usually occurs at much higher millimolar ranges in Caco-2 cells [33].

At the cellular level, 5-HMF may promote oxidative stress through redox imbalance and impairment of antioxidant defenses. Previous studies have shown that 5-HMF can react with cellular glutathione and affect cellular redox homeostasis. In gastrointestinal epithelial models, 5-HMF has also been reported to induce ROS production, apoptosis, tight junction disruption, and modulation of MAPK-related pathways [12,13,15]. ROS accumulation may then contribute to the activation of stress-related signaling pathways, including MAPK/ERK and NF-κB, which are closely linked to inflammatory responses.

Consistent with this evidence, we demonstrated that 5-HMF significantly increased intracellular ROS production, indicating oxidative stress in differentiated Caco-2 cells. This finding is consistent with previous studies showing that 5-HMF can exert harmful effects on gastrointestinal epithelial cells, including oxidative stress, apoptosis, tight junction disruption, inflammatory activation, and modulation of MAPK-related pathways [12,13]. Interestingly, HMF-induced ROS accumulation was not accompanied by significant modulation of NRF2 mRNA expression, suggesting that, under our experimental conditions, 5-HMF did not activate a compensatory NRF2-dependent transcriptional response. However, 5-HMF reduced GPX1 and GPX4 mRNA expression, while SOD1 remained unchanged, indicating a selective impairment of glutathione-dependent antioxidant defenses rather than a generalized suppression of antioxidant enzymes [34]. This is biologically relevant because GPX1 and GPX4 are key antioxidant enzymes involved in the detoxification of hydrogen peroxide and lipid hydroperoxides, respectively [35].

The reduction in GPX4 may be particularly relevant in the context of lipid-related epithelial stress. Although GPX4 has well-established tissue-specific functions, especially in the brain and testes, evidence indicates that it also plays an important role in intestinal epithelial cells. In Caco-2 cells, gastrointestinal glutathione peroxidases have been shown to limit lipid hydroperoxide transport [36], while GPX4 contributes to the protection of mitochondria from oxidative damage and to the maintenance of oxidative phosphorylation complexes in gut epithelial cells [37]. Moreover, impaired GPX4 activity and lipid peroxidation have been associated with intestinal inflammatory conditions [38]. Therefore, the reduction in GPX4 mRNA expression observed after 5-HMF exposure, together with increased ROS production and neutral lipid accumulation, may indicate an impaired capacity to detoxify lipid hydroperoxides in differentiated Caco-2 cells. However, since GPX4 protein levels, enzymatic activity, lipid peroxidation markers, and ferroptosis-specific endpoints were not assessed, these findings should be interpreted as evidence of altered antioxidant defense rather than direct evidence of ferroptosis [39].

The reduction in GPX4 may be particularly relevant in relation to lipid-related stress, as GPX4 protects cells from lipid hydroperoxide accumulation [39]. In parallel, 5-HMF increased neutral lipid accumulation and PLIN2 expression, suggesting altered lipid storage and lipid-related epithelial stress. Since PLIN2 is a lipid droplet-associated protein commonly used as a marker of intracellular neutral lipid storage [40], the parallel increase in Oil Red O staining and PLIN2 expression supports the hypothesis that 5-HMF promotes lipid droplet accumulation in differentiated Caco-2 cells. Importantly, blueberry juice reduced ROS production, increased NRF2 and attenuated GPX1 and GPX4 expression, and decreased both neutral lipid accumulation and PLIN2 levels. These findings support a protective role of blueberry juice against HMF-induced oxidative and lipid-related epithelial stress, in agreement with previous studies showing that blueberry-derived anthocyanins and polyphenols can reduce oxidative stress and modulate NRF2-related antioxidant pathways in intestinal epithelial models [19,41]. This biological protection is deeply rooted in the specific phytochemical profile of the blueberry juice we characterized. Spectrophotometric scanning of the juice revealed two prominent absorbance maxima at 293 nm and 520 nm, corresponding to the total phenolic fraction and anthocyanins, respectively, but affected by organic acids and other compounds. In fact, while isolated phenolic monomers typically exhibit an absorbance peak around 270–280 nm, the maximum detected at 293 nm reflects a clear bathochromic shift due to the complex effect of the food matrix. This phenomenon should be attributable to hydrophobic interactions and π-π stacking between juice molecules, thus altering the native UV-Vis spectrum. Furthermore, the matrix-induced effect is further reflected in the visible region above 500 nm, where it was not possible to notice a clear peak but a slight curve around ~520 nm. Our hypothesis is supported by data previously published [40,41,42] and further confirmed by our HPLC analyses, which identified catechin and cyanidin, representing approximately 18% of the total polyphenols, despite the presence of other unidentified peaks, which finally conferred on the blueberry juice an antioxidant activity of ~31%I. Overall, our data are also consistent with those previously described by Lohachoompol, Huang, and their collaborators [43,44].

Moreover, our data showed that 5-HMF increased NF-κB protein expression and the mRNA levels of NLRP3, IL1B, and IL18, suggesting the induction of a pro-inflammatory response in differentiated Caco-2 cells. This result is consistent with previous studies showing that 5-HMF can damage gastrointestinal epithelial cells. In particular, Qiu et al. reported that 5-HMF induced oxidative stress, apoptosis, and tight junction disruption in gastric mucosal epithelial cells, while Qin et al. showed that 5-HMF affected intestinal epithelial cells and modulated MAPK-related genes [12,13]. These studies support our observation that 5-HMF can promote epithelial stress and inflammatory activation. In contrast with our findings, Kong et al. showed that 5-HMF reduced LPS-induced inflammation in RAW 264.7 macrophages by inhibiting MAPK, NF-κB, and mTOR signaling [45]. Similarly, Zhang et al. reported that 5-HMF attenuated LPS-induced lung injury by suppressing the NF-κB/NLRP3 inflammasome pathway [46]. These apparently opposite results may be explained by the fact that the biological effects of 5-HMF appear to depend not only on dose, but also on cell type, inflammatory background, exposure time, and experimental context.

**Figure 7:**
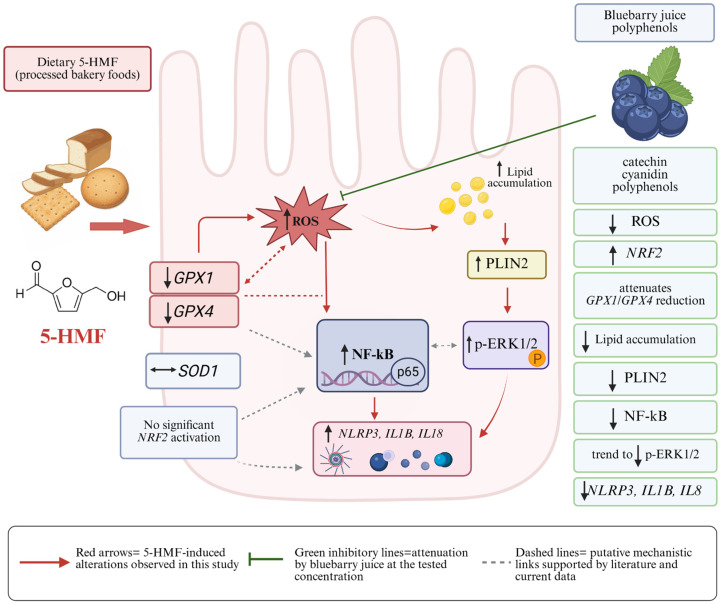
Proposed schematic representation of 5-HMF-induced epithelial stress and its attenuation by blueberry juice in differentiated Caco-2 cells. The scheme summarizes the relationships among the measured oxidative stress markers, lipid-related alterations, and inflammatory mediators. Red arrows indicate 5-HMF-induced alterations observed in this study; green inhibitory lines indicate the attenuating effects of blueberry juice co-treatment at the tested concentration; dashed lines indicate putative mechanistic links supported by the present findings and previous literature.

To better summarize these findings, we added a schematic representation illustrating the interrelationship among the measured oxidative, lipid-related, and inflammatory parameters, as well as the putative signaling pathways modulated by blueberry juice in 5-HMF-exposed differentiated Caco-2 cells (Figure 7).

This study has some limitations. First of all, despite the chemical characterization of blueberries, the absence of strong analytical techniques such as mass spectroscopy makes it impossible to completely determine all the biomolecules contained in blueberries. Concerning the in vitro studies, although differentiated Caco-2 cells are a widely used model of the intestinal epithelium, they do not fully reproduce the complexity of the human intestinal mucosa, including mucus-producing cells, immune cells, gut microbiota, digestive processes, and systemic metabolism. Secondly, the 5-HMF concentration used in vitro cannot be directly translated into human dietary exposure, as intestinal levels depend on digestion, food matrix interactions, dilution in gastrointestinal fluids, absorption, metabolism, and transit time. A further limitation is that oxidative and inflammatory endpoints were evaluated using a single 5-HMF concentration. Although MTT assays confirmed the absence of cytotoxicity at the tested concentrations, this experimental design does not allow assessment of dose-dependent oxidative stress responses. In addition, the inflammatory response was mainly assessed through NF-κB protein expression and the mRNA levels of NLRP3, IL1B, and IL18; therefore, these results support NLRP3-related inflammatory priming rather than complete inflammasome activation. Future studies should evaluate caspase-1 activation, ASC speck formation, and mature IL-1β/IL-18 secretion. Finally, blueberry juice was tested as a whole natural matrix; therefore, further analyses are needed to identify the specific bioactive compounds responsible for the observed protective effects. In addition, blueberry juice was evaluated at a single concentration, which does not allow assessment of dose-dependent effects or identification of the minimum effective concentration. Moreover, the individual effects of the main blueberry-derived phenolic compounds on 5-HMF-induced inflammatory mediators were not evaluated. In addition, experiments were performed only in differentiated Caco-2 cells and not in primary intestinal epithelial cells. Future studies should test the major identified compounds, alone and in combination, in both Caco-2 and primary intestinal epithelial models to better define their specific contribution and translational relevance.

This study has several strengths. First, it integrates food chemical analysis with an intestinal epithelial cell model, allowing the detection of 5-HMF in bakery products commonly consumed, especially in Western countries, to be linked with its potential biological effects at the gut level. Second, the use of HPLC-based quantification improved the reliability and specificity of 5-HMF detection in complex food matrices compared with colorimetric/spectrophotometric approaches. Third, differentiated Caco-2 cells were used as a mature enterocyte-like model to evaluate the effects of 5-HMF on intestinal epithelial stress.

## 4. Materials and Methods

### 4.1. Chemicals and Reagents

Hydroxymethylfurfural (5-HMF), resorcinol, hydrochloric acid (HCl), formic acid, methanol (MeOH), and acetonitrile (ACN) were purchased from Sigma-Aldrich (Merk Life Science S.r.l., Milan, Italy), as well as Folin–Ciocalteu reagent, sodium carbonate, sodium nitrite, aluminum chloride, gallic acid, 2,2-diphenyl-1-picrylhydrazyl (DPPH) and sodium hydroxide used for blueberry characterization (Merk Life Science S.r.l., Milan, Italy).

### 4.2. Food Matrix Preparation

Sweet and savory baked products, such as biscuits and crackers, were randomly purchased from local supermarkets and used as reference food matrices. Additional information, including food type, average cooking temperature and average cooking time, is listed in Supplemental Table S1. Bakery products were ground using a stainless-steel mortar and pestle. Powders obtained (1 g) were transferred into conical tubes and incubated with Milli-Q^®^ water at a 1:20 weight ratio. After vortexing, a solid–liquid extraction was performed using an ultrasonic bath (Vevor Digital Ultrasonic Cleaner – VEVOR - HomeBleve tool GmbH, Frankfurt am Main, Germany), set at 80 °C for 15 min. Ultrasound was applied for 1 min every 5 min intervals. Samples were centrifuged at 2500 rpm for 15 min at 4 °C, and supernatants were filtered through a 40 µm cellulose membrane. Extracts obtained were stored at 4 °C overnight prior to analysis.

Fresh and local blueberries were directly bought in a local store. Also in this case, blueberries were ground using a stainless-steel mortar and pestle and the juice obtained was centrifuged at 1500 rpm for 10 min at 4 °C, and the juice was filtered through 40 µm cellulose membrane filters before analysis.

### 4.3. Determination of 5-HMF in Bakery Products

The 5-HMF contained in bakery products was quantified by spectrophotometric and chromatographic approaches. The obtained analytical results were finally compared.

The UV–Vis spectrophotometer used was a ThermoScientific—Genesys^®^ 150 (ThermoScientific, Milan, Italy), equipped with Exacta Optec quartz cuvettes. Suitable calibration curves were obtained by using 5-HMF standard solutions (r^2^ ≥ 0.98 in all cases). All the analyses were conducted in triplicate, and appropriate dilutions were applied when necessary to maintain absorbance values within the linear range of the Lambert–Beer law. The colorimetric assay for 5-HMF quantification was performed as previously described by Geirola and coworkers [47]. Briefly, a working reagent consisting of 0.1% (*w*/*v*) resorcinol in 3% (*v*/*v*) HCl was prepared. Samples and reagent were mixed in a volume ratio of 1:4 and the mixture was incubated at 70 °C for 60 min. The reaction was stopped by incubating on ice. Absorbance (ABS) was measured in scan mode across all visible wavelengths (λ) and maximum values were selected at λ ≈ 484 nm.

The HPLC system consisted of a ThermoFisher Scientific Vanquish Core equipped with a quaternary pump, autosampler, thermostated column compartment, and UV–Vis detector (ThermoFisher Scientific—Rosano, Italy). Separations were performed on a reverse-phase C18 column (Acclaim^®^120, 4.6 × 100 mm, 5 µm particle size). The mobile phase consisted of 0.1% (*v*/*v*) formic acid in water and acetonitrile (ACN), with the organic phase increasing from 10% to 30% during the analysis. UV–Vis signals were acquired using dual-wavelength detection at 285 nm and 484 nm. Peak identification and quantification were performed by comparison with HMF standard solutions. Each calibration line was obtained with a concentration decrease of at least 5 points, and when possible, 8 points were used (r^2^ > 0.98). A volume of 10 µL of each sample was injected into the HPLC system.

### 4.4. Characterization of Blueberry Juice

Blueberry squeezed juice was analyzed with ThermoScientific-Genesys 150^®^ (ThermoFisher Scientific, Rosano, Italy) in 2 mL quartz cuvettes, as well as by HPLC. The spectrophotometer was set to scan mode with a wavelength (λ) range set between 240 nm and 700 nm. Subsequently, the juice was characterized in terms of total polyphenols content (TPC), total flavonoid content (TFC) and antioxidant activity, as previously described [48]. The total phenolic content was assessed by incubating the sample with Folin–Ciocalteu reagent diluted at a 1:30 ratio, according to the protocol recently published by Lawag and coworkers. Briefly, 200 µL of the sample was incubated with 1 mL of diluted Folin–Ciocalteu working reagent and 800 µL of sodium carbonate (0.7% *w*/*v*), because it proved to be particularly effective in carbohydrate-rich food matrices [49]. The obtained mixture was incubated in the dark for 120 min and absorbance was detected at 760 nm. Gallic acid solutions were used as reference standard molecule in concentrations between 0.032 ppm and 0.500 mg/mL (y = 4.804x + 0.006; r^2^ = 0.996). The results are listed in Table 2 as milligrams equivalent to gallic acid (mg GAE/mL). Total amount of flavonoids was assessed according to a recent protocol developed by Mare and coworkers [50]. Briefly, samples were incubated with sodium nitrite and aluminum chloride solutions. After the addition of sodium hydroxide solution, the absorbance was detected at 300 nm < λ < 400 nm. A mixture of flavonoids was used as reference standard molecules for obtaining a calibration curve with a good linear regression (r^2^> 0.98).

The antioxidant capacity of blueberry juice was assessed by evaluating the capacity of drupes to reduce free radicals in comparison to L-ascorbic acid. In detail, a 0.004% *w*/*v* solution of 2,2-Diphenyl-1-picrylhydrazyl (DPPH) was incubated in the dark for 30 min with blueberry juice. The absorbance was determined at λ = 517 nm, and the percentage of inhibition was calculated using the following equation:

I (%) = [(A_0_ − A_1_)/A_0_] × 100

A_0_ = absorbance of negative control; A_1_ = absorbance of extracts/standards.

Polyphenol profiling was conducted on a ThermoFisher Scientific Vanquish HPLC system operated through Chromeleon^®^ software (version 7.2). The instrumentation included a quaternary pump, autosampler, column oven, and UV/Vis detector, as mentioned above. A reversed-phase C18 column (100 × 4.6 mm, 5 μm; Phenomenex, Torrance, CA, USA), preceded by a 3 × 4.6 mm guard column, was utilized for separation. The elution gradient combined 10 mM aqueous H_3_PO_4_ and acetonitrile (ACN), increasing the latter from 15% to 40% (*v*/*v*) in 20 min, within a total analysis time of 25 min. Target analytes, including catechin and cyanidin, were detected at 270 nm. Standard molecules were used to build calibration curves ranging from 1.56 to 100 ppm. All the calibration models displayed high reliability, with linearity coefficients (r^2^) > 0.99 (Supplemental Figure S3).

### 4.5. Growth and Differentiation of Caco-2

The human colorectal adenocarcinoma cell line Caco-2, obtained from ATCC, was used in this study. Cells were cultured in Minimum Essential Medium (MEM) supplemented with 20% fetal bovine serum (FBS) and maintained at 37 °C in a humidified atmosphere containing 5% CO_2_. The culture medium was renewed twice weekly to maintain optimal growth conditions.

For differentiation into small intestinal enterocyte-like cells, Caco-2 cells were seeded in 6-well plates at a density of 2.5 × 10^5^ cells/well and cultured for 21 days, with medium replacement every 2 days, as previously described [51].

### 4.6. Treatments

After 21 days of differentiation, Caco-2 cells were treated for 48 h with 5-hydroxymethylfurfural (HMF). After 21 days of differentiation, Caco-2 cells were treated for 48 h with 5-HMF at a final concentration of 0.4 mM, either alone or in combination with blueberry juice. Blueberry juice was added at a final concentration corresponding to 15 µg/mL total phenolic equivalents.

### 4.7. Cell Viability

Cell viability was assessed using the MTT assay. For this assay, 1 × 104 cells/well were seeded in a 96-well dish and treated with 5-hydroxymethylfurfural (HMF) at concentrations of 0.2 mM or 0.4 mM, either alone or in combination with blueberry juice. Following treatment, 0.5 mg/mL of MTT solution was added, and the cells were incubated for another 4 h at 37 °C. The formazan crystals formed were then dissolved in 100 μL of dimethyl sulfoxide (DMSO). The absorbance was measured at 570 nm using a microplate reader (Microplate reader 800TS, BioTek Instruments, Inc., Winooski, VT, USA) to quantify the resulting color intensity.

### 4.8. Protein Extraction and Western Blotting

For protein analysis, Caco-2 cells were seeded in 35 mm culture dishes at a density of 5 × 10^5^ cells/dish. After 21 days of differentiation, cells were treated for 48 h as described above. At the end of the treatment period, cells were lysed using Mammalian Protein Extraction Reagent (M-PER; Pierce, Thermo Fisher Scientific).

Protein expression in cell lysates was analyzed by Western blot according to standard protocols. The following primary antibodies were used: rabbit anti-phospho-ERK1/2 (Thr202/Tyr204; #9101, Cell Signaling Technology, Danvers, MA, USA), rabbit anti-NF-κB p65, and mouse anti-β-ACTIN (#3700, Cell Signaling Technology, Danvers, MA, USA). β-actin was used as a loading control.

### 4.9. RNA Extraction and Real-Time PCR

Caco-2 cells were seeded in 35 mm culture dishes at a density of 5 × 10^5^ cells/dish and allowed to differentiate for 21 days before treatment. After 48 h of treatment, total RNA was extracted using the WizPrep™ Total RNA Mini Kit (Wizbiosolutions Inc., Seongnam, Republic of Korea) according to the manufacturer’s instructions. RNA concentration and purity were assessed using a NanoDrop™ One/OneC Microvolume UV-Vis Spectrophotometer (Thermo Fisher Scientific, Rosano, Italy).

Complementary DNA (cDNA) was synthesized from 1 µg of total RNA using the High-Capacity cDNA Reverse Transcription Kit (Applied Biosystems, Foster City, CA, USA). The gene expression levels of NLRP3, IL1B, IL18, NFE2L2/NRF2, PLIN2, SOD1, GPX4, GPX1 and ACTB were quantified by real-time PCR using SYBR^®^ Green PCR Master Mix (Bio-Rad Laboratories, Inc., Hercules, CA, USA) and gene-specific primers, as reported in Supplementary Table S1. Relative gene expression was calculated using the comparative 2^^−ΔΔCt^ method and normalized to ACTB expression (Supplemental Table S2).

### 4.10. Reactive Oxygen Species (ROS)

Intracellular ROS production was evaluated using the fluorogenic probe CellROX™ Green Reagent (Molecular Probes, Thermo Fisher Scientific, Rosano, Italy). Differentiated Caco-2 cells were treated for 48 h with HMF (384 µM), either alone or in combination with blueberry juice/extract (15 µg/mL). At the end of the treatment period, cells were incubated with 5 µM CellROX™ Green Reagent according to the manufacturer’s instructions. Cell nuclei were counterstained with DAPI (1 µg/mL). Fluorescence images were acquired at 40× magnification using an Axio Observer Imaging System equipped with AxioVision 3.1 software (Zeiss, Oberkochen, Germany).

### 4.11. Neutral Lipid Content

Intracellular neutral lipid accumulation was assessed by Oil Red O staining. Caco-2 cells were seeded in 60 mm culture dishes at a density of 5 × 10^5^ cells/dish and allowed to differentiate before treatment. After 48 h of treatment, cells were washed with PBS and fixed with 4% paraformaldehyde for 20 min at room temperature. Cells were then stained with Oil Red O working solution (Sigma-Aldrich, St. Louis, MO, USA) for 20 min. Nuclei were counterstained with DAPI for an additional 20 min. All the staining procedures were performed at room temperature and protected from direct light exposure. Images were acquired at 40× magnification using an Axio Observer Imaging System equipped with AxioVision 3.1 software (Zeiss). Neutral lipid accumulation was quantified as Oil Red O-positive area using ImageJ 11 software (Fiji, Human Technopole, Milan, Italy) and normalized to the number of DAPI-positive nuclei.

### 4.12. Statistical Analysis

All data describe the average of three independent experiments and are expressed as the mean ± standard deviation (SD) or percentage in weight and volume. Mean and SD concerning all the physicochemical analyses were calculated by using Chromeleon Chromatography Data System (CDS) (ThermoFisher Scientific—Rosano, Italy) and Microsoft^®^ ExcelTM (Microsoft Office360). HPLC, UV/VIS and in vitro data were analyzed with the GraphPad Prism 8.0 software using a two-tailed Student’s *t*-test for pairwise comparisons. All analytical data were compared in terms of distribution (Shapiro–Wilk Normality test) and correlation (Pearson test and ANOVA Mixed Effects Model) by GraphPad Prism 8.0 software. In any case, significant differences were assumed to be present at *p* < 0.05.

## 5. Conclusions

In conclusion, this study shows that 5-HMF is detectable in bakery products commonly consumed as breakfast or snack foods and that its accurate quantification in complex food matrices requires selective analytical methods, such as HPLC. In differentiated Caco-2 cells, 5-HMF induced early intestinal epithelial stress, characterized by increased ROS production, reduced expression of selected antioxidant defenses, increased neutral lipid accumulation and induction of NF-κB/NLRP3-related inflammatory markers. These findings suggest that 5-HMF may negatively affect intestinal epithelial homeostasis under high-exposure conditions. Importantly, blueberry juice attenuated most of the HMF-induced alterations, reducing oxidative stress, improving antioxidant responses, decreasing lipid accumulation and limiting inflammatory markers. These results suggest that fresh fruits rich in polyphenols and antioxidant compounds, such as blueberries, may help attenuate, at least in part, the epithelial stress induced by food-processing contaminants such as 5-HMF.

Overall, our findings provide preliminary in vitro evidence supporting the importance of limiting excessive consumption of heat-processed bakery products, particularly during childhood, while promoting the inclusion of fresh antioxidant-rich fruits within healthy dietary patterns. However, the possible protective effect of fruit consumption against HMF-related intestinal stress should be confirmed in future studies using digested food matrices, co-digestion models and more complex intestinal systems.

## Figures and Tables

**Table 1:** Chemical quantification of 5-HMF contained in bakery products.

	UV-VIS Spectrophotometer		HPLC			
	Colorimetric Assay		Colorimetric Assay		Traditional ANALYSIS	
#	ABS (λ = 484 nm)	ppm ± SD	ppm ± SD (λ = 484 nm)	mg/100 g ± DS	ppm ± SD (λ = 285 nm)	mg/100 g ± DS
1	0.46 ± 0.06	216.3 ± 26.9	n.a.	n.a.	0.045 ± 0.005	0.089 ± 0.010
2	0.41 ± 0.05	190.8 ± 24.7	3.65 ± 4.44	7.30 ± 8.88	0.019 ± 0.001	0.038 ± 0.003
3	0.56 ± 0.07	265.6 ± 31.5	5.82 ± 6.64	11.64 ± 13.29	0.315 ± 0.022	0.629 ± 0.044
4	0.58 ± 0	271.5 ± 0.2	5.11 ± 5.54	10.22 ± 11.08	0.147 ± 0.003	0.294 ± 0.006
5	0.67 ± 0.02	314.8 ± 10.9	5.27 ± 6.55	10.54 ± 13.11	0.456 ± 0.317	0.912 ± 0.634
6	0.49 ± 0.06	228.1 ± 26.6	n.a.	n.a.	0.042 ± 0.004	0.083 ± 0.007
7	0.47 ± 0.04	218.4 ± 19	3.36 ± 4.48	6.71 ± 8.96	0.013 ± 0.006	0.026 ± 0.011
8	0.541 ± 0.001	254 ± 0.5	3.25 ± 3.79	6.50 ± 7.58	0.272 ± 0.022	0.543 ± 0.044
9	0.54 ± 0.01	254.6 ± 2.8	n.a.	n.a.	0.038 ± 0.014	0.076 ± 0.028
10	1.01 ± 0.06	477 ± 29.1	8.09 ± 10.94	16.18 ± 21.89	0.641 ± 0.066	1.281 ± 0.132
11	0.49 ± 0.03	231.9 ± 15.6	3.38 ± 4.44	6.77 ± 8.88	0.041 ± 0.001	0.082 ± 0.003
12	0.51 ± 0.02	241.3 ± 8,6	n.a.	n.a.	0.049 ± 0.004	0.097 ± 0.007
13	−0.12 ± 0.01	−58.9 ± 5	0.346044	0.69	0.394 ± 0.032	0.787 ± 0.064
14	0.41 ± 0.14	193.9 ± 64.6	24.54 ± 16.25	49.08 ± 32.49	0.199 ± 0.009	0.397 ± 0.018
15	0.08 ± 0.15	32.7 ± 72.5	1.55 ± 1.24	3.10 ± 2.48	0.534 ± 0.076	1.068 ± 0.153
16	0.31 ± 0.18	146.6 ± 86	16.41 ± 23.10	32.81 ± 46.20	0.391 ± 0.078	0.782 ± 0.156
17	0.35 ± 0.21	163.8 ± 98.4	26.82 ± 21.18	53.64 ± 42.36	0.051 ± 0.003	0.102 ± 0.006
18	0.34 ± 0.24	159 ± 113.2	15.89 ± 16.36	31.77 ± 32.73	0.359 ± 0.002	0.717 ± 0.004
19	−0.06 ± 0.01	−32.7 ± 5.9	1.55 ± 1.28	3.09 ± 2.55	1.103 ± 0.006	2.205 ± 0.013
20	0.13 ± 0.05	60.6 ± 22.5	0.37 ± 0.29	0.74 ± 0.57	0.024 ± 0.001	0.048 ± 0.003
21	−0.07 ± 0.01	−35.1 ± 4.9	0.65 ± 0.13	1.29 ± 0.26	0.177 ± 0.032	0.353 ± 0.064
22	0.37 ± 0.08	174.2 ± 39.8	17.09 ± 16.86	34.18 ± 33.71	0.109 ± 0.001	0.218 ± 0.003
23	0.08 ± 0.02	35.6 ± 8.4	2.03 ± 0.17	4.06 ± 0.35	0.844 ± 0.091	1.687 ± 0.182
24	0.41 ± 0.19	193.5 ± 88.6	14.18 ± 9.67	28.36 ± 19.34	0.009 ± 0.001	0.018 ± 0.000
25	0.41 ± 0.29	194 ± 138.7	19.17 ± 13.94	38.34 ± 27.87	0.127 ± 0.023	0.253 ± 0.047
26	−0.03 ± 0	−19.1 ± 1	1.61 ± 0.42	3.21 ± 0.85	0.399 ± 0.021	0.798 ± 0.042

ABS = absorbance, ppm = parts per million; SD = standard deviation; n.a. = Not Available.

**Table 2:** Characterization of fresh squeezed blueberry juice.

Assay/Protocol	ABS ± SD	Concentration
TPC	0.286 ± 0.007	0.587 ± 0.015 mg GAE/mL
TFC	0.739 ± 0.075	44.30 ± 4.91 ppm
Antioxidant Activity	1.527 ± 0.078	30.95 ± 3.53%I

TPC = total phenolic content; TFC = total flavonoid content; mg GAE/mL = milligrams of gallic acid equivalents per milliliter; ABS = absorbance; ppm = parts per million; SD = standard deviation; %I = inhibition percentage.

## Data Availability

The original data presented in the study are openly available upon request from the journal Editor or the corresponding author.

## Supplementary Materials

The following supporting information can be downloaded at https://www.mdpi.com/article/10.3390/ijms27156665/s1.
