# Supplementary material for: Modulation of Dietary 5-Hydroxymethylfurfural-Induced Intestinal Epithelial Stress by Blueberry Polyphenols
Source: Int J Mol Sci. 2026 Jul 26;27(15):6665. doi: 10.3390/ijms27156665 (PMC13467082; doi:10.3390/ijms27156665)
Supplement: Supplementary file 1 [file ijms-27-06665-s001.zip › Supplemental Matherials.pdf]

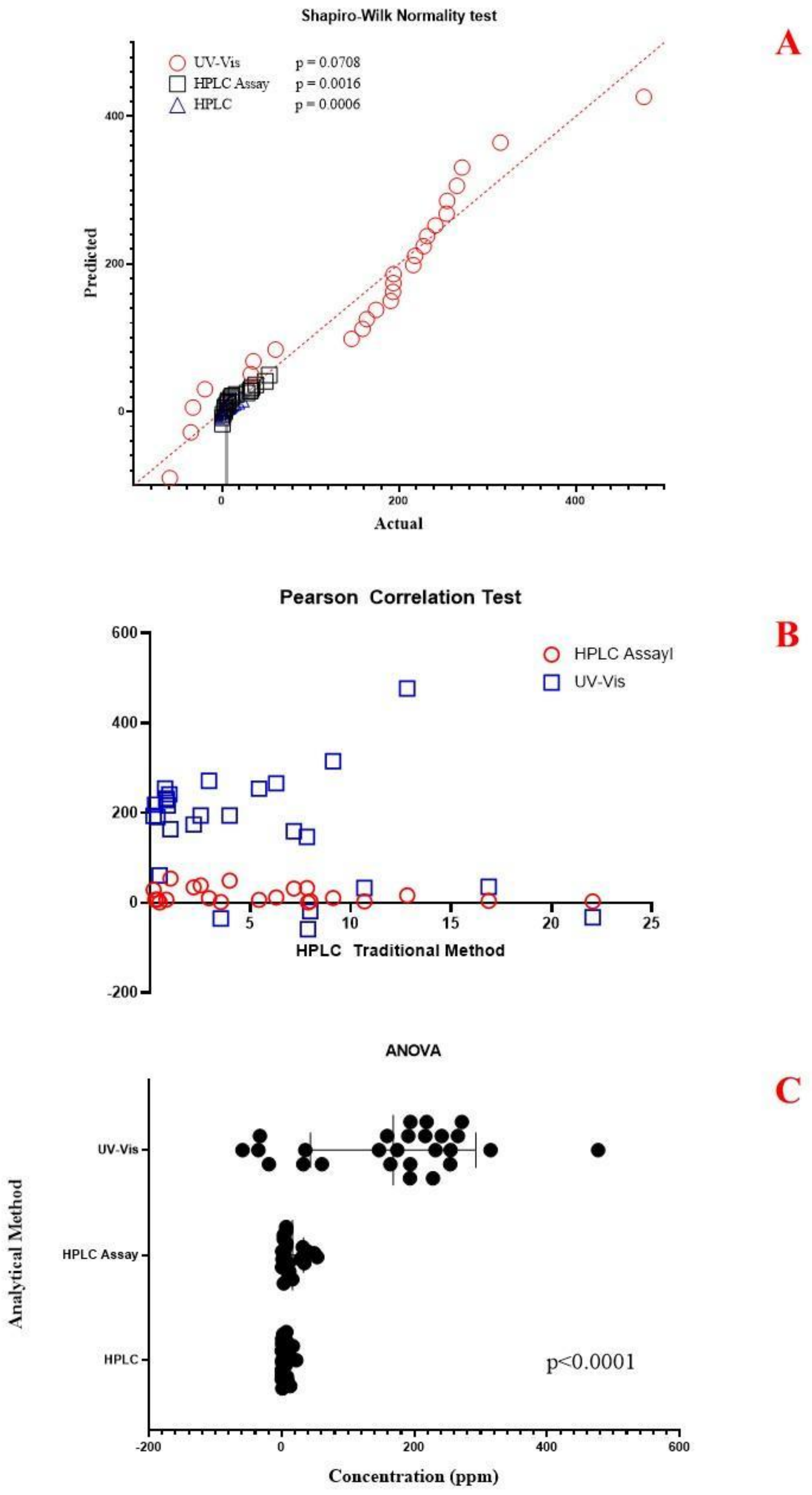

Figure S1. Comparative statistical evaluation of the three analytical methods for HMF quantification in bakery products. (A) Shapiro-Wilk normality test profiles for UV-Vis (red circles), HPLC Assay (black squares), and traditional HPLC (blue triangles), with corresponding p-values indicating data distribution normality. (B) Pearson correlation plots comparing the UV-Vis spectrophotometric method (blue squares) and the HPLC Assay (red circles) against the traditional HPLC reference approach. (C) Dot plot distribution of HMF concentrations (ppm) obtained by each analytical framework; data were analysed using a one-way ANOVA mixed-effects model ($p < 0.0001$).

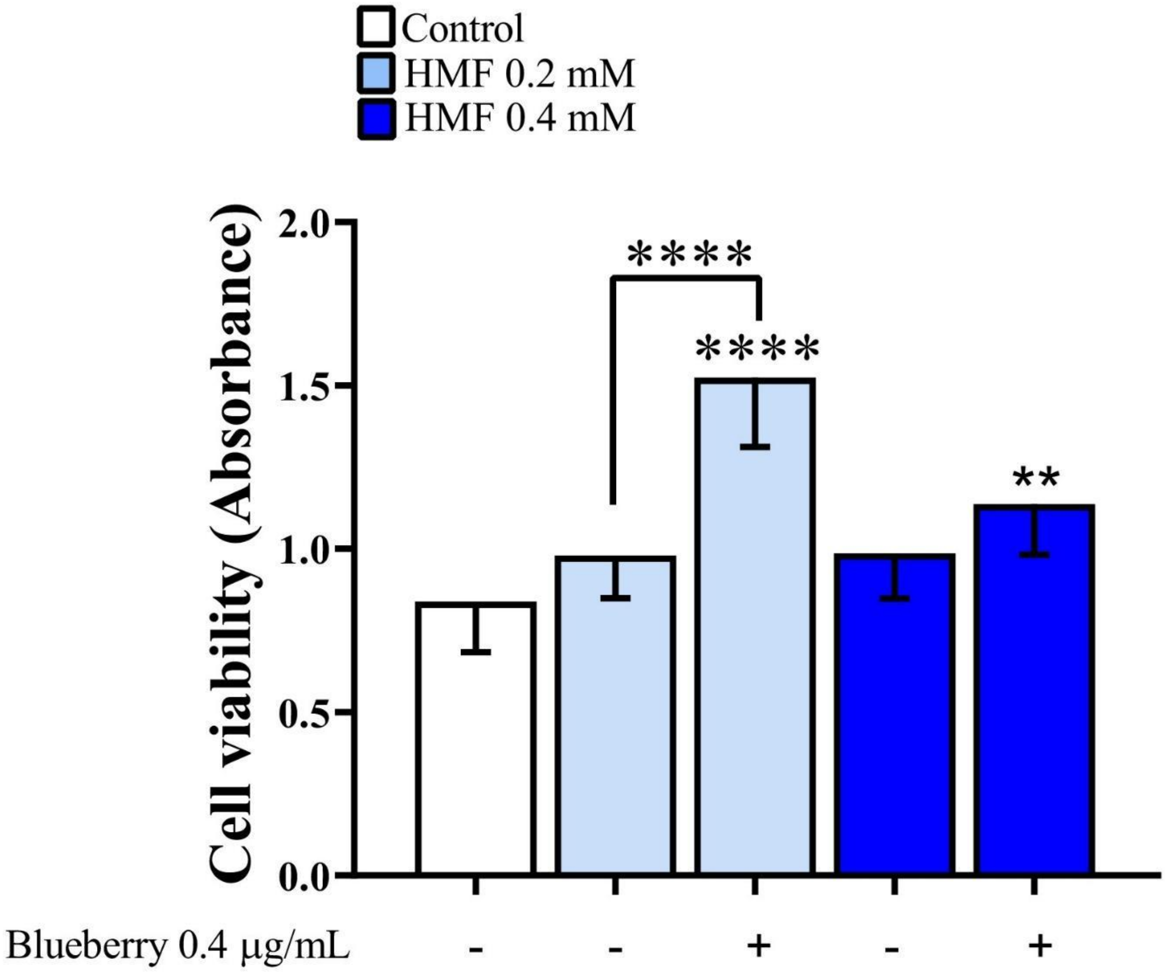

Supplemental figure S2: Effect of HMF (0.2 and 0.4 mM), alone or in combination with blueberry extract (0.4 µg/mL), on cell viability assessed by the MTT assay. Cells were treated with HMF (0.2 or 0.4 mM) in the absence or presence of blueberry extract (0.4 µg/mL). Cell viability is presented as mean ± SD of at least three independent experiments. Statistical analysis was performed using a Student's t-test. Significant differences are indicated as $P < 0.01$ (**) and $P < 0.0001$ (****).

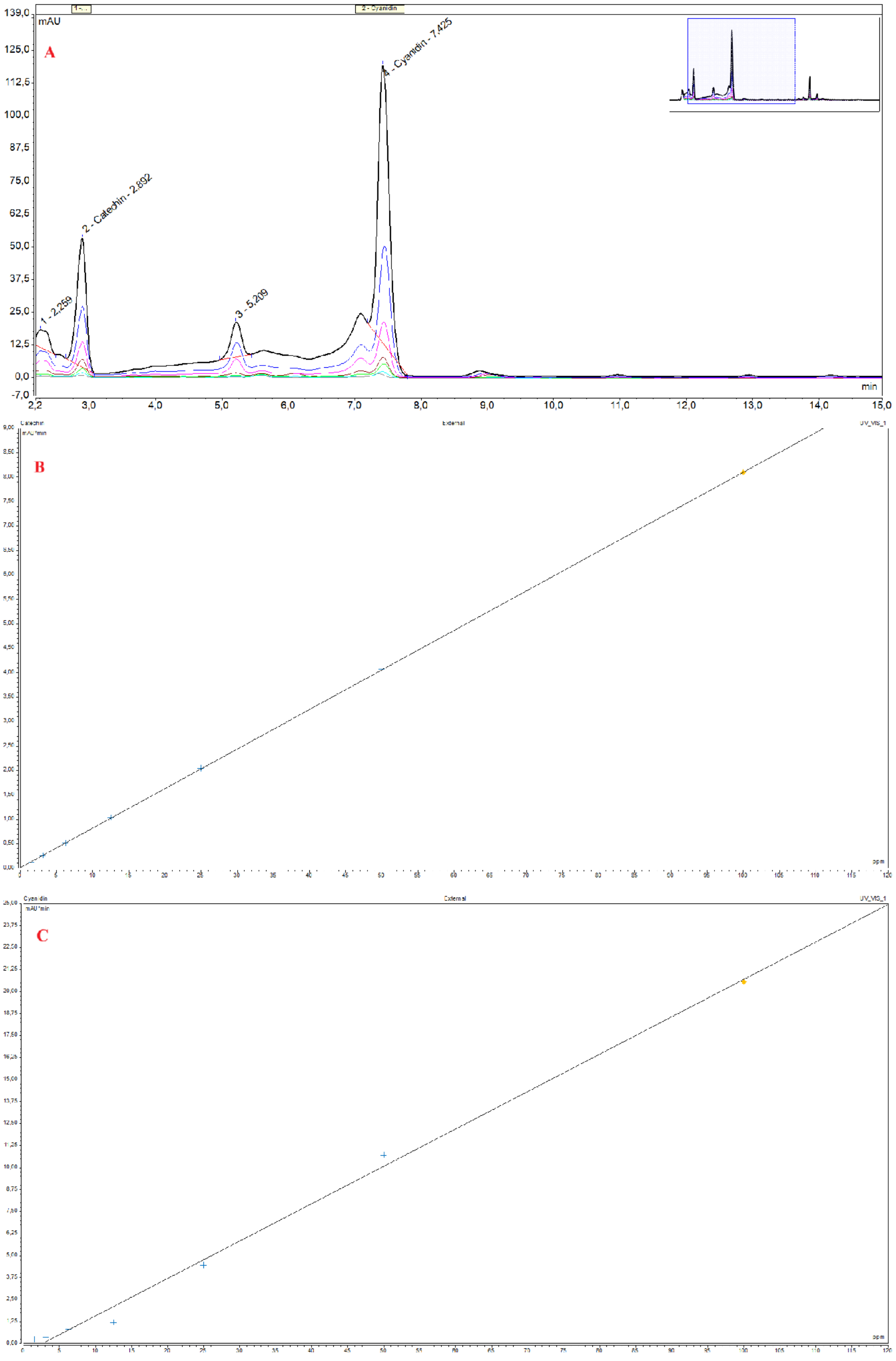

Figure S3. Chromatogram (A) obtained by HPLC analyses and calibration curves of standard molecules effectively quantified in blueberries, including catechin (B) and cyanidin (C).

Supplemental Table S1. Type of food and preparation/cooking characteristics.

N°	Food Type	Temperature (°C)	Average Cooking Time (min)
1	Cracker	200 - 220	8 – 12
2	Cracker	200 - 220	8 – 10
3	Biscuit / Cookie	170 - 180	12 – 15
4	Biscuit / Cookie	170 - 180	10 – 12
5	Biscuit / Cookie	170 - 180	12 – 15
6	Cracker	200 - 220	8 – 12
7	Cracker	200 - 220	8 – 10
8	Cracker	200 - 220	8 – 10
9	Biscuit / Cookie	170 - 180	12 – 15
10	Biscuit / Cookie	170 - 180	12 – 15
11	Cracker	200 - 220	8 – 10
12	Cracker	200 - 220	8 – 12
13	Bread Rusks	200 / 150	15 (Baking) / 20-30 (Toasting)
14	Biscuit / Cookie	170 - 180	13 – 16
15	Cracker	200 - 220	7 – 10
16	Biscuit / Cookie	170 - 180	10 – 13
17	Breakfast Cereal	140 - 150	15 - 20 (Toasting)
18	Biscuit / Cookie	170 - 180	12 – 15
19	Bread Rusks	200 / 150	12 (Baking) / 15-20 (Toasting)
20	Biscuit / Cookie	170 - 180	11 – 14
21	Cracker / Snack	200 - 220	10 – 12
22	Biscuit / Cookie	170 - 180	10 – 13
23	Cracker	200 - 220	9 – 12
24	Biscuit / Cookie	170 - 180	11 – 14
25	Biscuit / Cookie	170 - 180	12 – 15
26	Cracker / Snack	200 - 220	8 – 11

Table S2. List of genes analysed.

Gene	Forward 5’-3’	Reverse 5’-3’
hIL-1beta	ATGATGGCTTATTACAGTGGCAA	GTCGGAGATTCCGTAGCTGGA
hIL-18	AAACTATTTGTCGCAGGAATAAAGAT	GCTTGCCAAAGTAATCTGATTCC
hNLRP3	AAGGGCCATGGACTATTTCC	GACTCCACCCGATGACAGTT
hNRF2	CACATCCAGTCAGAAACCAGTGG	GGAATGTCTGCGCCAAAAGCTG
hSOD1	GCAGATGACTTGGGCAAAGG	TGGGCGATCCCAATTACACC
hPLIN2	ACAGGGGTGATGGACAAGAC	ATCATCCGACTCCCCAAGAC
hGPX1	CCCAAGCTCATCACCTGGTC	TGTCAATGGTCTGGAAGCGG
hGPX4	GAAATGCCATCAAGTGGAAC	CAGGTCCTTCTCTATCACCA
hACTB	GCCCTGAGGCACTCTTCCA	TTGCGGATGTCCACGTCA

hIL-1beta (interleukin 1 beta), **hIL-18** (interleukin 18), **hNLRP3** (NLR Family Pyrin Domain Containing 3), **hNRF2** (NFE2 Like BZIP Transcription Factor 2) beta actin (HUGO Gene Nomenclature Committee abbreviation ACTB/ACTB) **hGPX1** (Glutathione Peroxidase 1) hGPX4 (Glutathione Peroxidase 4) hSOD1 (Superoxide Dismutase 1), hPLIN2 (Perilipin 2) and ***hACTB (***beta actin)
